# Supplementary material for: Wet-dry cycles enable the parallel origin of canonical and non-canonical nucleosides by continuous synthesis
Source: Nat Commun. 2018 Jan 11;9:163. doi: 10.1038/s41467-017-02639-1 (PMC5765019; doi:10.1038/s41467-017-02639-1)
Supplement: Supplementary file 1 — Supplementary Information [file 41467_2017_2639_MOESM1_ESM.pdf]

Supplementary Methods

General Information

Chemicals were purchased from Sigma-Aldrich, Fluka, ABCR, Carbosynth or Acros organics and used without further purification. Solutions were concentrated *in vacuo* on a Heidolph rotary evaporator. The solvents were of reagent grade or purified by distillation. Chromatographic purification of products was accomplished using flash column chromatography on Merck Geduran Si 60 (40-63 μm) silica gel (normal phase). Thin layer chromatography (TLC) was performed on Merck 60 (silica gel F254) plates. Visualization of the developed chromatogram was performed using fluorescence quenching or standard staining solutions. ^{1}H- and ^{13}C-NMR spectra were recorded in deuterated solvents on Varian Oxford 200, Bruker ARX 300, Varian VXR400S, Varian Inova 400, Bruker AMX 600 and Bruker AVIIIHD 400 spectrometers and calibrated to the residual solvent peak. Multiplicities are abbreviated as follows: s = singlet, d = doublet, t = triplet, q = quartet, m = multiplet, br = broad. High-resolution ESI spectra were obtained on the mass spectrometers Thermo Finnigan LTQ FT-ICR. IR measurements were performed on Perkin Elmer Spectrum BX FT-IR spectrometer with a diamond-ATR (Attenuated Total Reflection) setup. Melting points were measured on a Büchi B-540. For preparative HPLC purification a Waters 1525 binary HPLC Pump in combination with a Waters 2487 Dual Absorbance Detector was used, with Macherey-Nagel VP 250/10 Nucleosil 100-7 C18 reversed phase column. The prebiotic reactions were analyzed by LC-ESI-MS on a Thermo Finnigan LTQ Orbitrap XL and were chromatographed by a Dionex Ultimate 3000 HPLC system with a flow of 0.15 mL/min over an Interchim Uptisphere120-3HDO C18 column. The column temperature was maintained at 30 °C. Eluting buffers were buffer A (2 mM $HCOONH_4$ in H_2O (pH 5.5)) and buffer B (2 mM $HCOONH_4$ in H_2O/MeCN 20/80 (pH 5.5)). The gradient for reactions of FaPy **5a-h** with ribose was 0 → 45 min; 0% → 20% or 0% → 10% buffer B. The elution was monitored at 260 nm (Dionex Ultimate 3000 Diode Array Detector). The chromatographic eluent was directly injected into the ion source without prior splitting. Ions were scanned by use of a positive polarity mode over a full-scan range of m/z 120-1000 with a resolution of 30000. Parameters of the mass spectrometer were tuned with a freshly mixed aqueous solution of adenosine (5 μM). The synthetic standards for the coinjection experiments were synthesized in our lab (see synthesis of the synthetic standards) or purchased from Sigma-Aldrich, Fluka, ABCR, Carbosynth or Acros organics. The X-ray intensity data was measured at a temperature of 100 K on a Bruker D8 Venture TXS system equipped with a multilayer mirror optics monochromator and a Mo Kα rotating-anode X-ray tube (λ = 0.71073 Å). The frames were integrated with the Bruker SAINT software package using a narrow-frame algorithm. Data were corrected for absorption effects using the Multi-Scan method (SADABS). The structures were solved and refined using the Bruker SHELXTL software package.

Synthetic Procedures

6-imino-1-methyl-5-nitroso-1,6-dihydropyrimidine-2,4-diamine (4a)

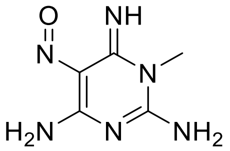

Synthetic reference: To 6-imino-1-methyl-1,6-dihydropyrimidine-2,4-diamine hydrogen iodide[1] (6.00 g, 22.5 mmol, 1 eq.) in H_2O was added CF_3COOAg (5.00 g, 22.5 mmol, 1 eq.). The flask was vigorously shaken for 5-10 min. The precipitate was filtered off over celite and washed with H_2O (15 mL). CF_3COOH (17.5 mL, 225 mmol, 10 eq.) was added to the filtrate and the solution was cooled in an ice/H_2O bath. $NaNO_2$ (1.63 g, 23.6 mmol, 1.05 eq.) dissolved in H_2O (15 mL) was added dropwise. After stirring for 30 min at room temperature, the formed precipitate was filtered off and washed with H_2O and Acetone (each 15 mL) to give 6-imino-1-methyl-5-nitroso-1,6-dihydropyrimidine-2,4-diamine triflouroacetic acid (4.13 g, 20.4 mmol, 65%). For comparison with the prebiotic product, a sample of the triflouroacetic acid salt was obtained as a free base by treatment with 3 M NH_4OH. After sonication the product was filtered off to give a reddish solid that turned pink again after drying.

^{1}H-NMR (400 MHz, DMSO-*d*$_6$) δ = 11.44 (s, 1H), 8.41 (s, 1H), 8.08 (br, 1H), 7.67 (br, 1H), 7.49 (s, 1H), 3.26 (s, 3H). **^{13}C-NMR** (101 MHz, DMSO-*d*$_6$) δ = 165.44, 157.64, 146.95, 137.22, 27.64. **HRMS** (ESI+): calc. for $[C_5H_9N_6O]^+$ 169.0832, found: 169.0832 $[M+H]^+$

2-(methylthio)-5-nitrosopyrimidine-4,6-diamine (4b)

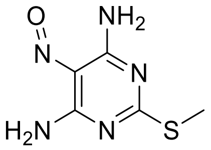

Synthetic reference: 2-(methylthio)pyrimidine-4,6-diamine (2.00 g, 12.8 mmol, 1 eq.) was dissolved in H_2O (50 mL) and AcOH (2.8 mL) and cooled in an ice/H_2O bath. It was added a solution of $NaNO_2$ (1.92 g, 27.8 mmol, 2.2 eq.) in H_2O (20 mL). The reaction was kept for 2 h at 0 °C and the turquoise precipitate was filtered off to give 2-(methylthio)-5-nitrosopyrimidine-4,6-diamine (2.20 g, 11.9 mmol, 93%).

^{1}H-NMR (400 MHz, DMSO-*d*$_6$) δ 10.18 (d, *J* = 4.2 Hz, 1H), 9.00 (s, 1H), 8.42 (d, *J* = 4.2 Hz, 1H), 8.02 (s, 1H), 2.46 (s, 3H). **^{13}C-NMR** (101 MHz, DMSO-*d*$_6$) δ 179.05, 164.73, 146.22, 139.43, 14.08. **HRMS** (ESI+): calc. for $[C_5H_8N_5OS]^+$ 186.0444, found: 186.0444 $[M+H]^+$

5-nitrosopyrimidine-2,4,6-triamine (4c)

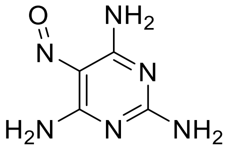

Synthetic reference: The reaction was performed slightly modified according to literature.[2] 6-aminopyrimidine-2,4-diamine (900 mg, 7.20 mmol, 1eq.) was suspended in H_2O (10 mL). AcOH (660 µl, 11.5 mmol, 1.6 eq.) was added and the reaction mixture cooled in an ice/H_2O bath. A solution of $NaNO_2$ (520 mg, 7.50 mmol, 1.05 eq.) in H_2O (3 mL) was added dropwise. After stirring for about 30 min at room temperature, the pink precipitate that formed was filtered off to give 5-nitrosopyrimidine-2,4,6-triamine (987 mg, 6.40 mmol, 89%).

^{1}H-NMR (400 MHz, DMSO-d_6) δ = 10.26 (d, J = 5.1 Hz, 1H), 8.15 (s, 1H), 7.75 (d, J = 5.1 Hz, 1H), 7.35 (s, 1H), 7.19 (s, 2H). **^{13}C-NMR** (101 MHz, DMSO-d_6) δ = 166.52, 165.32, 151.43, 138.04. **HRMS** (ESI+): calc. for $[C_4H_7N_6O]^+$ 155.0676, found: 155.0676 $[M+H]^+$

2-methyl-5-nitrosopyrimidine-4,6-diamine (4d)

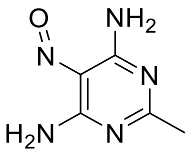

Synthetic reference: Acetamidine (**2d**) salt of (hydroxyimino)malononitrile (**3**) (2.00 g, 13.5 mmol) was heated in 5-Ethyl-2-methylpyridine (10 mL) at 180°C for 20 min. After cooling to room temperature, the mixture was diluted with EtOH. After standing the product precipitated as green solid that was filtered off to give 2-methyl-5-nitrosopyrimidine-4,6-diamine (1.70 g, 11.5 mmol, 85%).

^{1}H-NMR (400 MHz, DMSO-d_6) δ = 10.04 (d, J = 3.2 Hz, 1H), 8.97 (s, 1H), 8.36 (d, J = 3.2 Hz, 1H), 7.96 (s, 1H), 2.20 (s, 3H). **^{13}C-NMR** (101 MHz, DMSO-d_6) δ = 175.20, 166.38, 146.76, 139.94, 26.83. **HRMS** (ESI-): calc. for $[C5H6N5O]^-$ 152.0578, found: 152.0578 $[M-H]^-$

6-amino-2-(methylthio)-5-nitrosopyrimidin-4(3H)-one (4e)

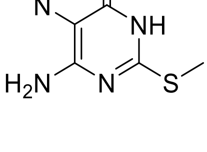

Prebiotic Synthesis: 2-(methylthio)-5-nitrosopyrimidine-4,6-diamine (1.00 g, 5.40 mmol, 1 eq.) was stirred in HCl (6 M, 500 mL) for 7 days at 0-8 °C. The sample was filtered and freeze dried and the yellow residue was taken up in a minimal amount of H_2O. The color of the residue changed to blueish/violet immediately and was filtered off to give 6-amino-2-(methylthio)-5-nitrosopyrimidin-4(3H)-one (760 mg, 4.10 mmol, 76%).

For analytic reasons 20 mg of the sample was purified by dissolving it in NH_4OH (30%, 3 mL). AcOH was slowly dropped into the solution until pH ~8-9 was reached. The product crystalized and was filtered off to give pink colored shiny crystals (12.0 mg). These crystals are treated with a minimal volume of AcOH (10%). The now blueish/violet crystals are filtered off. The sample is NMR clean but shows still 15% of starting material.

Synthetic standard: 6-amino-2-(methylthio)pyrimidin-4(3H)-one (1.00 g, 6.40 mmol, 1 eq.) was dissolved in H_2O containing NaOH (210 mg, 5.30 mmol, 0.8 eq.). To the dissolved pyrimidine was added $NaNO_2$ (480 mg, 7.00 mmol, 1.1 eq.). The solution was acidified with AcOH (660 µl, 11.6 mmol, 1.8 eq.), causing immediately a white precipitate that turned blue overnight upon standing. The blue precipitate was filtered off to give 6-amino-2-(methylthio)-5-nitrosopyrimidin-4(3H)-one (850 mg, 4.60 mmol, 72%).

^{1}H-NMR (400 MHz, DMSO-d_6) δ = 12.72 (s, 1H), 11.26 (s, 1H), 9.08 (s, 1H), 2.53 (s, 3H). **^{13}C-NMR** (101 MHz, DMSO-d_6) δ = 168.88, 161.57, 147.29, 143.44, 13.52. **HRMS** (ESI-): calc. for $[C_5H_5N_4O_2S]^-$ 185.0139, found: 185.0138 $[M-H]^-$

2,6-diamino-3-methyl-5-nitrosopyrimidin-4(3H)-one (4f)

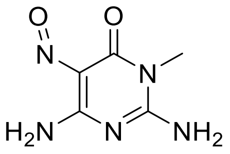

Prebiotic synthesis: 6-imino-1-methyl-5-nitroso-1,6-dihydropyrimidine-2,4-diamine (500 mg, 3.00 mmol, 1eq.) was dissolved in 0.5 M HCl (50 mL) and stirred at room temperature overnight. The reaction mixture was neutralized with NH_4OH upon which an reddish precipitate formed immediately. The reddish product was filtered off after cooling to give 2,6-diamino-3-methyl-5-nitrosopyrimidin-4(3H)-one (330 mg, 1.95 mmol, 65% over 2 steps from 1-methylguanidine (**2a**) salt of (hydroxyimino)malononitrile (**3**)).

The yield of **4f** could only be determined over 2 steps, because we used impure **4a** directly from the prebiotic reaction. Therefore, the yield from **4a** to **4f** was determined by using synthetic

6-imino-1-methyl-5-nitroso-1,6-dihydropyrimidine-2,4-diamine triflouro-acetic acid (500 mg, 1.80 mmol) that was dissolved in 0.5 M HCl (30 mL) and stirred at room temperature overnight. The reaction mixture was neutralized with NH_4OH upon which a pink precipitate formed immediately. The pink product was filtered off after cooling to give 2,6-diamino-3-methyl-5-nitrosopyrimidin-4(3H)-one (295 mg, 1.74 mmol, 97%).

Synthetic standard: 6-amino-2-methoxy-3-methyl-5-nitrosopyrimidin-4(3*H*)-one (2.50 g, 13.6 mmol) was suspended in 20% NH_4OH (35 mL) and stirred at room temperature for 1 hour and 45 min. The pink solid was collected by filtration and washed with H_2O, EtOH and Et_2O, to give 2,6-diamino-3-methyl-5-nitrosopyrimidin-4(3H)-one (2.07 g, 12.2 mmol, 90%).

^{1}H-NMR (400 MHz, DMSO-*d*$_6$) δ = 10.83 (d, *J* = 4.9 Hz, 1H), 8.25 (s, 1H), 8.07 (d, *J* = 4.9 Hz, 1H), 7.80 (s, 1H), 3.32 (s, 3H). **^{13}C-NMR** (101 MHz, DMSO-*d*$_6$) δ = 162.10, 157.33, 151.10, 142.39, 28.35. **HRMS** (ESI+): calc. for $[C_5H_8N_5O_2]^+$ 170.0673, found: 170.0672 $[M+H]^+$

6-amino-2-(methylamino)-5-nitrosopyrimidin-4(3H)-one (4g)

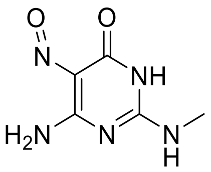

6-amino-2-(methylthio)-5-nitrosopyrimidin-4(3H)-one (**4e**, 400 mg, 2.16 mmol, 1 eq.) was dissolved in $MeNH_2$ (aq.) (1%, 21 mL) and stirred for 4 h at 8°C. The color changed from blueish/violet to dark red. The mixture was then neutralized with AcOH. The product precipitated as an orange/brownish solid and was filtered off to give 6-amino-2-(methylamino)-5-nitrosopyrimidin-4(3H)-one (258 mg, 1.52 mmol, 70%).

To determine the yield for the transformation of **4e** to **4g** from completely pure material, synthetic 6-amino-2-(methylthio)-5-nitrosopyrimidin-4(3H)-one (100 mg, 0.54 mmol, 1 eq.) was dissolved in $MeNH_2$ (aq.) (1%, 6 mL) and stirred for 4 h at 8°C. The color changed from violet to dark red. The mixture was then neutralized with AcOH. The product precipitated as an orange solid and was filtered off to give 6-amino-2-(methylamino)-5-nitrosopyrimidin-4(3H)-one (84 mg, 0.50 mmol, 93%).

Due to insolubility, a **^{13}C-NMR** was not possible **^{1}H-NMR** (400 MHz, DMSO-*d*$_6$) δ = 11.25 (s, 1H), 10.90 (s, 1H), 8.53 (s, 1H), 7.21 (q, *J* = 4.7 Hz, 1H), 2.85 (d, *J* = 4.7 Hz, 4H). **HRMS** (ESI+): calc. for $[C_5H_8N_5O_2]^+$ 170.0673, found: 170.0672 $[M+H]^+$

6-amino-2-(dimethylamino)-5-nitrosopyrimidin-4(3H)-one (4h)

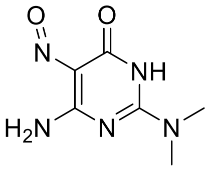

6-amino-2-(methylthio)-5-nitrosopyrimidin-4(3H)-one (**4e**, 350 mg, 1.90 mmol, 1 eq.) was dissolved in Me_2NH (aq.) (1%, 21 mL) and stirred for 4 h at 8°C. The color changed from blueish/violet to dark red. The mixture was then neutralized with AcOH. The product precipitated to form a reddish/pinkish paste like mixture. The precipitate was filtered off to give 6-amino-2-(methylamino)-5-nitrosopyrimidin-4(3H)-one (256 mg, 1.46 mmol, 73%).

To determine the yield for the transformation of **4e** to **4h** from completely pure material, synthetic 6-amino-2-(methylthio)-5-nitrosopyrimidin-4(3H)-one (1.00 g, 5.40 mmol, 1 eq.) was stirred in Me_2NH (aq.) (1%, 60 mL) for 4 h at 8°C. The mixture was then neutralized with AcOH. The product precipitated to form a pink paste like mixture. The precipitate was filtered off to give 6-amino-2-(dimethylamino)-5-nitrosopyrimidin-4(3H)-one (790 mg, 4.30 mmol, 80%). If desired, the product can be recrystallized from H_2O (50 mg in ca. 10 mL)

^{1}H-NMR (400 MHz, DMSO-d_6) δ = 10.96 (s, 2H), 8.29 (d, J = 5.2 Hz, 1H), 3.13 (s, 6H). **^{13}C-NMR** (101 MHz, DMSO-d_6) δ = 163.01, 154.49, 151.52, 141.75, 37.93. **HRMS** (ESI+): calc. for $[C_6H_{10}N_5O_2]^+$ 184.0829, found: 184.0829 $[M+H]^+$

2,6-diamino-5-nitrosopyrimidin-4(3H)-one (4i)

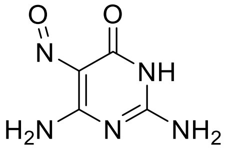

From synthetic 6-amino-2-(methylthio)-5-nitrosopyrimidin-4(3H)-one (**4e**): (40 mg, 0.21 mmol, 1 eq.) was heated in NH_4OH (2 M, 3 mL) for 3 days at 60 °C. The suspension was neutralized with AcOH and the product was filtered off, to give pink 2,6-diamino-5-nitrosopyrimidin-4(3H)-one (25.0 mg, 0.16 mmol, 76%).

From 5-nitroso-1,6-dihydropyrimidine-2,4,6-triamine (**4c**): (500 mg, 3.25 mmol, 1 eq.) was suspended in HCl solution (0.5 M, 50 mL) and stirred overnight at room temperature. The mixture was neutralized with NH_4OH and the product was filtered off, to give 2,6-diamino-5-nitrosopyrimidin-4(3H)-one (450 mg, 2.90 mmol, 89%).

Note: In case the conversion is inefficient at room temperature, the solution can also be heated up to 90°C.

NMR and ESI experiments are not possible due to insolubility even in warm DMSO. Therefore the product was confirmed by further reaction to the FaPyG compound as described below. **IR** (cm^{-1}): 3284 (s), 3151 (s), 3111 (s), 1701 (s), 1625 (s), 1580 (w), 1497 (m), 1459 (m), 1359 (m), 1314 (s), 1258 (s), 1145 (s), 1032 (m), 988 (m), 810 (m) 782 (s), 733 (m), 702 (m), 683 (s). **HRMS** (EI+): calc. for $[C_4H_5N_5O_2]^+$ 155.0438, found: 155.0436 $[M]^+$

General procedure for the formation and isolation of FaPy compounds from nitrosopyrimidines under prebiotic conditions (5a-h)

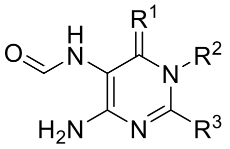

The corresponding nitrosopyrimidine (250 mg) was suspended in dilute HCOOH (20%, 30 mL) in water in the presence of elementary Ni powder (1.00 g). (Note: all tranformations except for **5b** were also done with Fe powder) The reaction mixture was stirred at 70 °C in a sealed 100 mL Ace pressure tube. The reaction time is indicated below for the corresponding compound. The solvent was then evaporated completely until dryness. The remaining solid was taken up in water (50 mL) and the pH was adjusted with solid K_2CO_3 until pH 9-10. Precipitation occurred immidiately. The mixture was further stirred for about 30 min at rt and the precipitate filtered off through celite. Note: if the pH is < 8 after filtration add some more K_2CO_3 and filter again. The clear solution is then concentrated (usually to about <1/10 of the volume) until the product precipitated. After cooling the product was filtered off to give the yield as indicated below. All FaPy compounds can be recrystallized from H_2O (50-100 mg in 5 mL of H_2O depending on the FaPy compound).

***N*-(2,4-diamino-1-methyl-6-oxo-1,6-dihydropyrimidin-5-yl)formamide (FaPym^{1}G, 5a)**

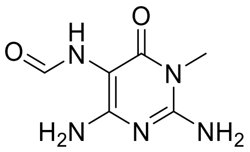

The nitrosopyrimidine **4f** (250 mg, 1.48 mmol) was reacted for 45 min. Isolated yield: 137 mg (0.75 mmol, 51%). The NMR shows cis/trans rotamers (~1.5:1)

^{1}H-NMR (400 MHz, DMSO-*d*$_6$) δ = 8.54 (d, cis, *J* = 1.6 Hz, 1H; NH), 8.02 (d, cis, *J* = 1.6 Hz, 1H; CHO), 7.93 (d, trans, *J* = 11.7 Hz, 1H; NH), 7.73 (d, trans, *J* = 11.7 Hz, 1H; CHO), 6.78 (s, trans, 2H; C2NH$_2$), 6.71 (s, cis, 2H; C2NH$_2$), 5.92 (s, trans 2H; C4NH$_2$), 5.69 (s, 2H; C4NH$_2$), 3.17 (s, cis/trans, 6H; 2 CH$_3$). **^{13}C-NMR** (101 MHz, DMSO-*d*$_6$) δ = 167.45 (trans, CHO), 161.07

(cis, CHO), 160.82 (trans, C6), 160.12 (cis, C6), 159.43 (trans, C4), 158.55 (cis C4), 154.45 (trans, C2), 154.22 (cis, C2), 88.98 (trans, C5), 88.94 (cis, C5), 28.35 (trans, CH_3), 28.19 (cis, CH_3). **HRMS** (ESI+): calc. for $[C_6H_{10}N_5O_2]^+$ 184.0829, found: 184.0830 $[M+H]^+$.

N-(4,6-diamino-2-(methylthio)pyrimidin-5-yl)formamide (FaPyms^{2}A, 5b)

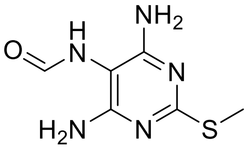

The nitrosopyrimidine **4b** (250 mg, 1.35 mmol) was reacted for 2 h in the presence of Ni. Isolated yield: 148 mg (0.74 mmol, 55%, over 2 steps from ethylthioamidine (**2b**) salt of (hydroxyimino)malononitrile (**3**)). The NMR shows cis/trans rotamers (~5:1).

^{1}H-NMR (400 MHz, DMSO-*d*$_6$) δ = 8.66 (s, cis, 1H; NH), 8.23 (d, trans, *J* = 11.5 Hz, 1H; NH), 8.07 (s, cis, 1H; CHO), 7.74 (d, trans, *J* = 11.3 Hz, 1H; CHO), 6.18 (s, trans, 4H: 2 NH_2), 6.01 (s, cis, 4H; 2 NH_2), 2.35 (s, cis/trans, 6H, 2 CH_3). **^{13}C-NMR** (101 MHz, DMSO-*d*$_6$) δ = 167.46 (trans, C2), 166.92 (cis, C2), 166.25 (trans, CHO), 161.52 (cis, CHO), 161.09 (trans, C4 and C6), 159.77 (cis, C4 and C6), 91.39 (trans, C5), 91.00 (cis, C5), 13.78 (cis/trans, 2 CH_3). **HRMS** (ESI+): calc. for $[C_6H_9N_5OS]^+$ 200.0601, found: 200.0600 $[M+H]^+$.

N-(4,6-diaminopyrimidin-5-yl)formamide (FaPyA, 5c)

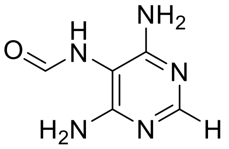

The nitrosopyrimidine **4b** (500 mg, 2.70 mmol) was reacted for 2 h. **5b** was the only product. Isolated yield of **5b**: 300 mg (1.50 mmol, 56%). The isolated **5b** (250 mg, 1.35 mmol, 1 eq.) was directly reacted under the same conditions for 7 d. H_2 was bubbled through the mixture before the start of reaction. **5c** was the only product. Isolated yield of **5c**: 106 mg (0.69 mmol, 51%). The NMR shows cis/trans rotamers (~5:1).

The overall yield without **5b** as isolated intermediate was determined by using synthetic nitrosopyrimidine **4b** (250 mg, 1.35 mmol) that was reacted for 7 d in the presence of Ni. H_2 is additionally bubbled through the solution for 2-3 minutes before the start of reaction. Isolated yield: 95 mg (0.62 mmol, 46%).

^{1}H-NMR (400 MHz, DMSO-*d*$_6$) δ = 9.01 (s, cis, 1H; NH), 8.40 (d, trans, *J* = 11.5 Hz, 1H; NH), 8.09 (s, cis, 1H; CHO), 7.77 (d, trans, *J* = 11.5 Hz, 1H; CHO), 7.75 (s, trans, 1H; C2H), 7.74

(s, cis, 1H; C2H), 6.17 (s, trans, 4H; $C4NH_2$ and $C6NH_2$), 5.99 (s, cis, 4H; C4NH2 and C6NH2). **^{13}C-NMR** (101 MHz, DMSO-d_6) δ = 166.02 (trans, CHO), 161.28 (cis, CHO; trans C4 and C6), 159.86 (cis, C4 and C6), 156.46 (trans, C2), 156.08 (cis, C2), 94.54 (trans, C5), 94.37 (cis, C5). **HRMS** (ESI+): calc. for: $[C5H8N5O]^+$ 154.0723, found: 154.0725 $[M+H]^+$

***N*-(4-amino-2-(methylamino)-6-oxo-1,6-dihydropyrimidin-5-yl)formamide (FaPym^{2}G, 5d)**

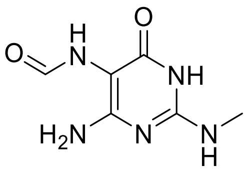

The nitrosopyrimidine **4g** (250 mg, 1.48 mmol) was reacted for 1 h. Isolated yield: 134 mg (0.74 mmol, 50%). The NMR shows cis/trans rotamers (~1.4:1)

^{1}H-NMR (400 MHz, DMSO-d_6) δ = 10.11 (s, cis/trans, 2H; $NH_{arom.}$), 8.49 (d, cis, *J* = 1.6 Hz, 1H; NH), 8.00 (d, cis *J* = 1.6 Hz, 1H; CHO), 7.86 (d, trans, *J* = 11.7 Hz, 1H; NH), 7.73 (d, trans, *J* = 11.7 Hz, 1H; CHO), 6.19 (br s, 1H; N$\underline{H}CH_3$), 6.14 (q, cis, *J* = 4.7 Hz, 1H, N$\underline{H}CH_3$), 6.08 (s, trans, 1H; NH_2), 5.87 (s, cis, 2H; NH_2), 2.73 (d, cis/trans, *J* = 4.7 Hz, 6H; 2 CH_3). **^{13}C-NMR** (101 MHz, DMSO-d_6) δ = 167.33 (trans, CHO), 161.60 (cis, C6), 161.24 (trans, C6), 161.10 (cis, CHO), 160.12 (cis, C4), 160.09 (trans, C4), 153.31 (trans, C2), 153.21 (cis, C2), 89.03 (trans, C5), 88.91 (cis, C5), 27.83 (cis/trans, 2 CH_3). **HRMS** (ESI+): calc. for $[C_6H_{10}N_5O_2]^+$ 184.0829, found: 184.0830 $[M+H]^+$.

***N*-(4-amino-2-(dimethylamino)-6-hydroxypyrimidin-5-yl)formamide (FaPym$^2{}_2$G, 5e)**

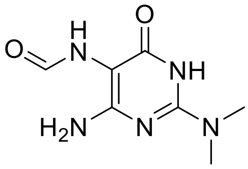

The nitrosopyrimidine **4h** (250 mg, 1.37 mmol) was reacted for 1 h. Isolated yield: 108 mg (0.55 mmol, 40%). The NMR shows cis/trans rotamers (~1.7:1).

^{1}H-NMR (800 MHz, DMSO-d_6) δ = 10.23 (br, cis/trans, 2H; $NH_{arom.}$), 8.54 (d, cis, *J* = 1.7 Hz, 1H; NH), 8.00 (d, cis, *J* = 1.7 Hz, 1H; CHO), 7.87 (d, trans, *J* = 11.7 Hz, 1H; NH), 7.74 (d, trans, *J* = 11.7 Hz, 1H; CHO), 6.05 (s, trans, 2H; NH_2), 5.83 (s, cis, 2H; NH_2), 2.98 (s, cis/trans, 12H; 4 CH_3). **^{13}C-NMR** (201 MHz, DMSO-d_6) δ 167.29 (trans, CHO), 161.96 (trans, C6), 161.02 (cis, C6), 161.00 (cis, CHO), 160.81 (trans, C4), 159.50 (cis, C4), 153.03 (cis/trans, C2), 88.70 (trans, C5), 88.67 (cis, C5), 37.52 (cis/trans, 4 CH_3). **HRMS** (ESI-): calc. for $[C_7H_{10}N_5O_2]^-$ 196.0840, found: 196.0840 $[M-H]^-$.

***N*-(2,4-diamino-6-oxo-1,6-dihydropyrimidin-5-yl)formamide (FaPyG, 5f)**

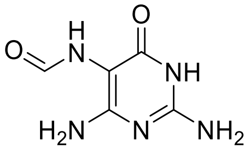

The nitrosopyrimidine **4i** (250 mg, 1.61 mmol) was reacted for 1.5 h. Isolated yield: 153 mg (0.91 mmol, 57%). The NMR shows cis/trans rotamers of the formamide group (~1.4:1).

^{1}H-NMR (400 MHz, DMSO-d_6) δ = 10.52 (s, cis/trans, 2H; $NH_{arom.}$), 8.50 (d, cis, J = 1.6 Hz, 1H; NH), 7.99 (d, cis, J = 1.6 Hz, 1H; CHO), 7.85 (d, trans, J = 11.7 Hz, 1H; NH), 7.72 (d, trans, J = 11.7 Hz, 1H; CHO), 6.47 (s, trans, 2H; C2NH_2), 6.36 (s, cis, 2H; C2NH_2), 5.98 (s, trans, 2H; C4NH_2), 5.75 (s, cis, 2H; C4NH_2). **^{13}C-NMR** (101 MHz, DMSO-d_6) δ = 166.96 (trans, CHO), 161.50 (trans, C6), 161.30 (cis, CHO), 160.60 (cis, C6), 160.18 (trans, C4), 159.90 (cis, C4), 154.00 (trans, C2), 153.88 (cis, C2) , 88.72 (cis/trans, C5). **HRMS** (ESI-): calc. for $[C_5H_6N_5O_2]^-$ 168.0527, found: 168.0527 $[M-H]^-$.

***N*-(2,4,6-triaminopyrimidin-5-yl)formamide (FaPyDA, 5g)**

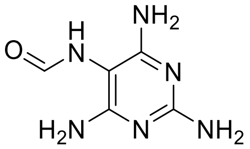

The nitrosopyrimidine **4c** (250 mg, 1.62 mmol) was reacted overnight. Isolated yield: 144 mg (0.86 mmol, 53%, over 2 steps from guanidine (**2b**) salt of (hydroxyimino)malononitrile (**3**)). The NMR shows cis/trans rotamers of the formamide group (~1.6:1).

^{1}H-NMR (400 MHz, DMSO-d6) δ = 8.49 (s, cis, 1H; NH), 8.06 (d, trans J=11.7, 1H; NH), 8.05 (s, cis, 1H; CHO), 7.69 (d, trans J=11.7, 1H; CHO), 5.68 (s, trans, 2H; C2NH_2), 5.53 (s, trans, 4H; C4NH_2 and C6NH_2), 5.51 (s, cis, 4H; C4NH_2 and C6NH_2), 5.43 (s, cis, 2H; C2NH_2) **^{13}C-NMR** (101 MHz, DMSO-d_6) δ = 166.57 (trans, CHO), 161.67 (trans, C4 and C6), 161.23 (cis, CHO), 161.14 (trans, C2), 161.09 (cis, C2), 160.13 (cis, C4 and C6), 86.79 (trans, C5), 86.54 (cis, C5). **HRMS** (ESI+): calc. for: $[C5H9N6O]^+$ 169.0832, found: 169.0832 $[M+H]^+$.

***N*-(4,6-diamino-2-methylpyrimidin-5-yl)formamide (FaPym^{2}A, 5h)**

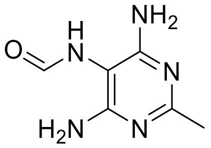

The nitrosopyrimidine (250 mg, 1.63 mmol) was reacted for 30 min. Isolated yield: 173 mg (1.04 mmol, 64%, over 2 steps from acetamidine (**2b**) salt of (hydroxyimino)malononitrile (**3**)). The NMR shows cis/trans rotamers (~5:1).

^{1}H-NMR (400 MHz, DMSO-d_6) δ = 8.68 (s, cis, 1H; NH), 8.24 (d, trans, *J* = 11.6 Hz, 1H; NH), 8.07 (d, cis, *J* = 1.2 Hz, 1H; CHO), 7.73 (d, trans, *J* = 11.6 Hz, 1H; CHO), 6.04 (s, trans, 4H, 2 NH_2), 5.86 (s, cis, 4H; 2 NH_2), 2.12 (s, cis/trans, 6H; 2 CH_3). **^{13}C-NMR** (101 MHz, DMSO-d_6) δ = 166.17 (trans, CHO), 164.70 (trans, C2), 164.23 (cis, C2), 161.35 (cis, CHO), 161.29 (trans, C4 and C6), 159.89 (cis, C4 and C6), 92.44 (trans, C5), 92.16 (cis, C5), 25.38 (cis/trans, 2 CH_3). **HRMS** (ESI+): calc. for $[C_6H_9N_5O]^+$ 168.0880, found: 168.0879 $[M+H]^+$.

Prebiotic nucleoside formation procedure (6a-h)

Nucleoside formation using ribose and FaPy (**5a-h**)

Ribose (56.5 mg, 0.375 mmol, 15 eq.) was thoroughly ground up with the corresponding FaPy compound **5a-h** (0.025 mmol, 1 eq.) and heated to 100 °C for 8 h in an oven. The remaining reaction mixture was taken up in basic solution (3 mL, 0.5 M Et_3N) and heated in a sealed tube (ACE 15 mL pressure tube) at 100 °C for several days (see below). 100 µL aliquots were removed and diluted to 1 mL. The aliquots were used for LC-MS analysis.

5a: reacted for 7d. **5b:** reacted for 14d**. 5c:** reacted for 21d. **5d:** reacted for 4d. **5e:** reacted for 4d. **5f:** reacted for 2d. **5g:** reacted for 21d. **5h:** reacted for 28d.

Synthesis of synthetic nucleoside standards

(2R,3R,4R,5R)-2-(acetoxymethyl)-5-(2-amino-6-oxo-1,6-dihydro-9H-purin-9-yl)tetrahydrofuran-3,4-diyl diacetate (7)

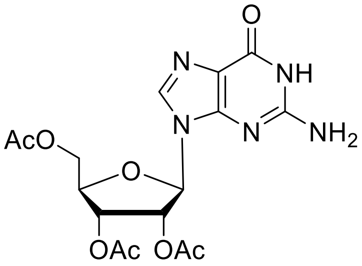

The reaction was carried out slightly modified to Robins *et al.*.[3,4]

Acetic anhydride (20 mL, 211 mmol, 12 eq.) was added to a mixture of guanosine (5.00 g, 18.6 mmol, 1 eq.) in DMF (12 mL) and pyridine (10 mL). After stirring for 1.5 h at 75 °C ethanol was added to quench the reaction. The mixture was filtered, the solvent removed *in vacuo* and the residue was recrystallized from isopropanol. The crystals were washed with cold isopropanol in order to obtain the desired product as a white solid (6.37 g, 15.6 mmol, 88%).

^{1}H-NMR (400 MHz, d6-DMSO) δ = 10.73 (s, 1H, NH), 7.93 (s, 1H, HC_{Ar}), 6.54 (s, 2H,NH_2), 5.98 (d, *J* =6.1 Hz, 1H, HC1’), 5.78 (t, *J*=6.1 Hz, 1H, HC2’), 5.49 (dd, *J* =5.9 Hz, *J* =4.0 Hz, 1H, HC3’), 4.72–4.08 (m, 3H, HC4’, HC5’), 2.11 (s, 3H, $COCH_3$), 2.04 (s, 3H, $COCH_3$), 2.03 (s, 3H, $COCH_3$). **^{13}C-NMR** (101 MHz, d6-DMSO) δ = 170.13 (CH3CO), 169.48 (CH3CO), 169.31 (CH3CO), 156.64 (C6), 153.91 (C2), 151.13 (C4), 135.65 (C8), 116.82 (C5), 84.36 (C1’), 79.55 (C4’), 72.04 (C2’), 70.32 (C2’), 63.10 (C5’), 20.57 (CH3CO), 20.42 (CH3CO), 20.22 (CH3CO). **HRMS** (ESI^+): calc. for $[C_{16}H_{20}N_5O_8]^+$ 410.1306, found: 410.1307 $[M+H]^+$.

(2R,3R,4R,5R)-2-(acetoxymethyl)-5-(2-amino-6-chloro-9H-purin-9-yl)tetrahydrofuran-3,4-diyl diacetate (8)

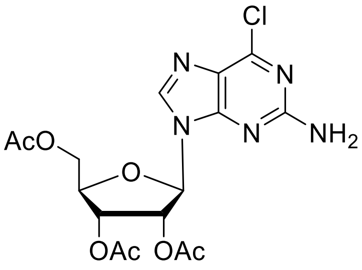

The reaction was carried out slightly modified to Robins *et al.*.[3,4]

Compound **7** (1.00 g, 2.44 mmol, 1 eq.) was dissolved in 80 mL dry acetonitrile. Triethylamine hydrochloride (0.81 g, 4.89 mmol, 2 eq), *N,N*-dimethylamine (0.31 mL, 2.44 mmol, 1 eq.) and phosphoryl chloride (1.37 mL, 14.66 mmol, 6 eq.) were added to the solution and the reaction mixture was heated to 100°C for 15 min. The solvent was removed *in vacuo* and the residue was dissolved in 30 mL water. The aqueous phase was extracted with chloroform. The combined organic layers were washed with $NaHCO_3$ and dried over $MgSO_4$ before the solvent was removed in vacuo. The crude product was purified by column chromatography (DCM : MeOH, 20:1) to afford the product as a white solid (0.56 g, 1.32 mmol, yield: 54%).

^{1}H-NMR (400 MHz, d_6-DMSO) δ = 8.36 (s, 1H, HC8), 7.08 (s, 2H, NH_2), 6.10 (d, ^3J=5.9 Hz, 1H, HC1’), 5.87 (t, *J*=5.9 Hz, 1H, HC2’), 5.53 (dd, *J*= 5.9 Hz, *J*=4.2 Hz, 1H, HC3’), 4.48 – 4.18 (m, 3H, HC4’, H2C5’), 2.12 (s, 3H, $COCH_3$), 2.03 (s, 3H, $COCH_3$), 2.03 (s, 3H, $COCH_3$). **^{13}C-NMR** (101 MHz, d_6-DMSO) δ = 170.52 ($COCH_3$), 169.84 ($COCH_3$), 169.71 ($COCH_3$), 160.04 (C2), 153.87(C6), 150.03 (C4), 141.28 (C8), 123.42 (C5), 84.85 (C1’), 79.70 (C4’), 71.89 (C2’),

70.25 (C3'), 62.97(C5'), 20.55 ($COCH_3$), 20.41 ($COCH_3$), 20.22 ($COCH_3$). **HRMS** (ESI+): calc. for $[C_{16}H_{19}ClN_5O_7]^+$ 428.0968, found: 428.0969 $[M+H]^+$.

(2R,3R,4R,5R)-2-(acetoxymethyl)-5-(6-chloro-2-iodo-9H-purin-9-yl)tetrahydrofuran-3,4-diyl diacetate (9) [5]

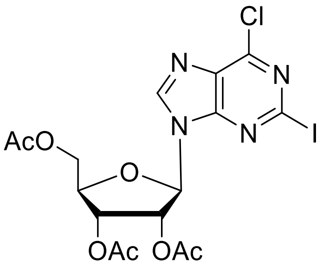

Nucleoside **8** (390 mg, 0.91 mmol, 1 eq), CuI (190 mg, 0.99 mmol, 1.1 eq.) and I_2 (230 mg, 0.91 mmol, 1 eq), was dissolved in dry THF (4.5 mL). CH_2I_2 (0.73 mL, 9.07 mmol, 10 eq.), and isoamyl nitrite (318 mg, 2.72 mmol, 3 eq.) were added and the solution was heated to 70°C. After 3 h the solvent was removed and the crude product was purified by column chromatography (DCM : MeOH, 200:1) to afford the product as a white solid (0.56 g, 1.32 mmol, yield: 54%).

^{1}H-NMR (400 MHz, d_6-DMSO) δ = 8.82 (s, 1H, HC8), 6.30 (d, ^3J = 4.9 Hz, 1H, HC1'), 5.89 (dd, ^3J = 5.9, 4.9 Hz, 1H, HC2'), 5.63 (t, J = 5.4 Hz, 1H, HC3'), 4.51 – 4.36 (m, 3H, H5', HC4'), 2.12 (s, 3H, $COCH_3$), 2.06 (s, 3H, $COCH_3$), 2.01 (s, 3H, $COCH_3$). **^{13}C-NMR** (101 MHz, d_6-DMSO) δ = 169.81 (COCH3), 169.66 (COCH3), 169.14 (COCH3), 151.98(C2), 148.79 (C6), 145.79 (C4), 131.45 (C8), 118.28 (C5), 84.85 (C1'), 86.00 (C4'), 69.59 (C2'), 69.42 (C3'), 62.42 (C5'), 20.38 ($COCH_3$), 20.18 ($COCH_3$), 20.05 ($COCH_3$). **HRMS** (ESI+): calc. for $[C_{16}H_{17}ClIN_4O_7]^+$ 538.9825, found: 538.9839 $[M+H]^+$.

(2R,3R,4R,5R)-2-(acetoxymethyl)-5-(6-chloro-2-methyl-9H-purin-9-yl)tetrahydrofuran-3,4-diyl diacetate (10)

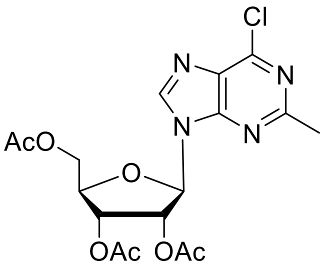

The reaction was carried out slightly modified to Yamazaki *et al.*.[6]

MeMgCl (2.56 M in THF, 0.22 mL, 573 µmol, 3 eq.) was added to $ZnCl_2$ (1.0 M in Et_2O, 0.29 mL, 286 µmol, 1.5 eq.) and) at 0°C and stirred for 3 h. This solution was diluted with THF (1mL) and added to a solution of **9** (103 mg, 192 µmol, 1 eq.) and $Pd(PPh_3)_4$ (22.1 mg, 19.1 µmol, 0.1 eq.) in dry THF (1.5 mL). The mixture was stirred for 10 min at 0°C and then at rt for additional 2 h. H_2O (10 mL) and DCM (5 mL) were added to the mixture. The phases were separated and the aqueous phase was extracted with DCM. The combined organic layers were washed with brine and dried over $MgSO_4$. The solvent was removed and the crude product was purified by column chromatography (DCM : MeOH, 20:1 → 10:1) to afford the product as a colorless foam (43.0 mg, 101 µmol, yield: 53%).

^{1}H-NMR (400 MHz, $CDCl_3$) δ = 8.12 (s, 1H, HC8), 6.12 (d, J = 4.9 Hz, 1H, HC1'), 5.89 (dd, ^3J = 5.9, 4.9 Hz, 1H, HC2'), 5.65 (t, J = 5.1 Hz, 1H, HC3'), 4.47 – 4.25 (m, 3H, HC4', H2C5'), 2.74 (s, 3H, $C_{Ar}CH_3$), 2.09 (s, 3H, $COCH_3$), 2.03 (s, 6H, $COCH_3$). **^{13}C-NMR** (101 MHz, $CDCl_3$) δ = 170.43 ($COCH_3$), 169.70 ($COCH_3$), 169.49 ($COCH_3$), 163.25 (C2), 151.85 (C4), 151.16 (C6), 143.03 (C8), 130.24 (C5), 86.90 (C1'), 80.49 (C4'), 73.20 (C2'), 70.61 (C3'), 63.11 (C5'), 25.86 ($CArCH_3$), 20.87 ($COCH_3$), 20.69 ($COCH_3$), 20.55 ($COCH_3$). **HRMS** (ESI+): calc. for $[C_{17}H_{20}ClN_4O_7]^+$ 427.1015, found: 427.1024 $[M+H]^+$.

(2R,3R,4S,5R)-2-(6-amino-2-methyl-9H-purin-9-yl)-5-(hydroxymethyl)tetrahydrofuran-3,4-diol (m^{2}A, 11)

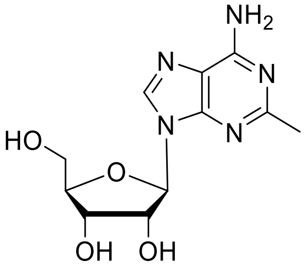

The reaction was carried out slightly modified to Yamazaki *et al.*.[7,8]

Nucleoside **10** (10.0 mg, 23.4 µmol, 1 eq.) was weighed into a pressure tube, into which ammonia was condensed. Subsequently the reaction mixture was stirred for 24 h at rt. Volatile compounds were removed in vacuo and the crude product was purified by HPLC to afford the product as a white solid (3.00 mg, 10.7 µmol, yield: 46%).

HPLC: Gradient: 100% HPLC buffer C, 0% HPLC buffer D → 80% HPLC buffer C, 20% HPLC buffer D in 45 min, retention time: 26.21 min, flow: 5 mL/min, column: VP 250/10 Nucleodur 100-5 C18ec.

^{1}H-NMR (400 MHz, CD_3OD) δ = 8.20 (s, 1H, HC8), 5.91 (d, J = 6.9 Hz, 1H, HC1'), 4.78 (dd, J = 6.8, 5.1 Hz, 1H HC2'), 4.31 (dd, J = 5.1, 1.0 Hz, HC3'), 4.18 (q, J = 2.1 Hz, 1H, HC4'), 3.90 (dd, ^{3}J = 12.6, 2.3 Hz, 1H, HaC5'), 3.74 (dd, J = 12.6, 2.3 Hz, 1H, HbC5'), 2.49 (s, 3H, $C_{Ar}CH_3$). **^{13}C-NMR** (101 MHz, CD_3OD) δ = 163.19 (C2), 150.36 (C6), 149.12 (C4), 141.71 (C8), 119.13 (C5), 91.37(C1'), 88.35 (C4'), 74.95 (C2'), 72.80 (C3'), 63.56 (C5'), 24.82 ($C_{Ar}CH_3$). **HRMS** (ESI+): calc. for $[C_{11}H_{16}N_5O_4]^+$ 282.1197, found: 282.1198 $[M+H]^+$.

(2S,3R,4R,5R)-2-(6-chloro-2-iodo-9H-purin-9-yl)tetrahydro-2H-pyran-3,4,5-triyl triacetate (12)

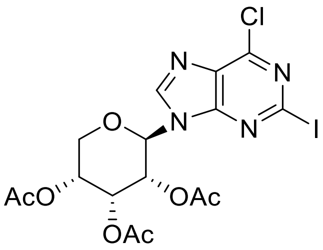

The reaction was carried out slightly modified to Vorbrüggen *et al.*.[9]

6-chloro-2-iodo-9H-purine (0.15 g, 0.53 mmol, 1.0 eq.) and acetylribose (323 mg, 1.02 mmol, 1.9 eq.) were suspended in dry acetonitrile (5.0 mL). *N*,*O*-Bis-(trimethylsilyl)-acetamide (195 mg, 0.96 mmol, 1.8 eq.) was added and the solution was heated to 60°C. After 30 min trimethylsilyltrifluoromethansolfonate (220 mg, 0.11 mmol, 2.0 eq.) was added and the solution turned from colorless to yellow-brown. The reaction mixture was cooled to rt, diluted with EtOAc and the organic layer was washed with $NaHCO_3$. The aqueous phase was washed with EtOAc and the combined organic layers were dried over $MgSO_4$. The solvent was removed and the crude product was purified by column chromatography (Hex : EtOAc, 1:1 → 1:4) to afford the product as a colorless foam (0.13 g, 0.23 mmol, yield: 44%).

^{1}H-NMR (400 MHz, $CDCl_3$) δ = 8.31 (s, 1H, HC8), 5.98 (d, J = 9.7 Hz, 1H, HC1'), 5.92 – 5.63 (m, 1H, HC2'), 5.50 (dd, J = 9.7, 2.9 Hz, 1H, HC3'), 5.18 (ddd, J = 10.5, 6.0, 2.7 Hz, 1H H2C5'), 4.31 – 3.71 (m, 1H H2C4'), 2.26 (s, 3H, $COCH_3$), 2.03 (s, 3H, $COCH_3$), 1.80 (s, 3H, $COCH_3$). **^{13}C-NMR** (101 MHz, $CDCl_3$) δ = 170.40 ($COCH_3$), 169.82 ($COCH_3$), 169.42 ($COCH_3$), 149.88 (C2), 149.33 (C4), 149.22 (C6), 143.01 (C8), 123.58 (C5), 78.86 (C1'), 68.10 (C3'), 68.00 (C2'), 65.83 (C4'), 63.92 (C5'), 21.03 ($COCH_3$), 20.72 ($COCH_3$), 20.43 ($COCH_3$). **HRMS** (ESI+): calc. for $[C_{16}H_{17}ClIN_4O_7]^+$ 538.9825, found: 538.9835 $[M+H]^+$.

(2S,3R,4R,5R)-2-(6-chloro-2-methyl-9H-purin-9-yl)tetrahydro-2H-pyran-3,4,5-triyl triacetate (13)

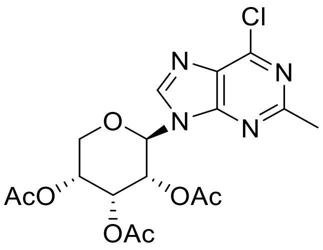

The reaction was carried out slightly modified to Yamazaki *et al.*.[6]

MeMgCl (2.56 M in THF, 0.22 mL, 557 µmol, 3 eq) was added to $ZnCl_2$ (1.0 M in Et_2O, 0.28 mL, 278 µmol, 1.5 eq.) and) at 0°C and stirred for 3 h. The solution was diluted with THF (1mL) and added to a solution of **12** (100 mg, 186 µmol, 1 eq.) and $Pd(PPh_3)_4$ (18.6 mg, 18.6 µmol, 0.1 eq.) in dry THF (1.5 mL). The mixture was stirred for 10 min at 0°C and then at rt for additional 2 h. H_2O (10 mL) and DCM (5 mL) were added to the mixture. The phases were separated and the aqueous phase was extracted with DCM. The combined organic layers were washed with brine and dried over $MgSO_4$. The solvent was removed and the crude product was purified by column chromatography (DCM : MeOH, 100:1) to afford the product as a colorless foam (36.0 mg, 84.4 µmol, yield: 45%).

^{1}H-NMR (400 MHz, $CDCl_3$) δ = 8.15 (s, 1H, HC8), 6.00 (d, J = 9.6 Hz, 1H, HC1'), 5.86 (td, J = 2.9, 0.9 Hz, 1H, HC2'), 5.73 (dd, J = 9.7, 2.8 Hz, 1H, HC3'), 5.25 (ddd, J = 10.5, 6.0, 2.8 Hz, 1H, C4'), 4.14 – 3.96 (m, 2H, H2C5'), 2.28 (s, 3H, $COCH_3$), 2.06 (s, 3H, $COCH_3$), 1.80 (s, 3H, $COCH_3$). **^{13}C-NMR** (101 MHz, $CDCl_3$) δ = 170.40 ($COCH_3$), 169.82 ($COCH_3$), 169.42 ($COCH_3$), 149.88 (C2), 149.33 (C4), 149.22 (C6), 143.01 (C8), 123.58 (C5), 78.86 (C1'), 68.10 (C3'), 68.00 (C2'), 65.83 (C4'), 63.92 (C5'), 21.03 ($COCH_3$), 20.72 ($COCH_3$), 20.43 ($COCH_3$). **HRMS** (ESI+): calc. for $[C_{17}H_{20}ClIN_4O_7]^+$ 427.1015, found: 427.1020 $[M+H]^+$.

(2R,3R,4R,5R)-2-(6-amino-2-methyl-9H-purin-9-yl)tetrahydro-2H-pyran-3,4,5-triol (*p*-m^{2}A, 14)

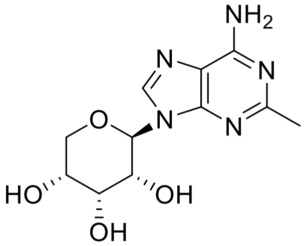

The reaction was carried out slightly modified to Yamazaki *et al.*.[7,8]

Nucleoside **13** (16.0 mg, 37.5 µmol, 1 eq.) was weighed into a pressure tube into which ammonia was condensed. Subsequently the reaction mixture was stirred for 24h at rt. Volatile compounds were removed *in vacuo* and the crude product was purified by HPLC to afford the product as a white solid (6.0 mg, 21.3 µmol, yield: 57%).

HPCL: Gradient: 100% HPLC buffer C, 0% HPLC buffer D → 80% HPLC buffer C, 20% HPLC buffer D in 45 min, retention time: 20.60 min, flow: 5 mL/min, column: VP 250/10 Nucleodur 100-5 C18ec.

^{1}H-NMR (400 MHz, CD_3OD) δ = 8.22 (s, 1H, HC8), 5.78 (d, ^3J = 8.7 Hz, 1H, HC1'), 4.26-4.22 (m, 2H, HC2'), 3.92-3.88 (m, 2H, H2C5'), 3.80-3.75 (m, 1H, C4'), 2.51 (s, 3H, $C_{Ar}CH_3$). **^{13}C-NMR** (101 MHz, CD_3OD) δ = 163.77 (C2), 156.95 (C6), 151.98 (C4), 140.90 (C8), 118.11 (C5), 81.67 (C1'), 72.81 (C4'), 70.38 (C2'), 68.18 (C3'), 66.60 (C5'), 25.27 ($C_{Ar}CH_3$).**HRMS** (ESI+): calc. for $[C_{11}H_{16}N_5O_4]^+$ 282.1197, found: 282.1198 $[M+H]^+$.

9-((3aR,4R,6R,6aR)-6-(hydroxymethyl)-2,2-dimethyltetrahydrofuro[3,4-d][1,3]dioxol-4-yl)-2-(((p-tolylthio)methyl)amino)-1,9-dihydro-6H-purin-6-one (15)

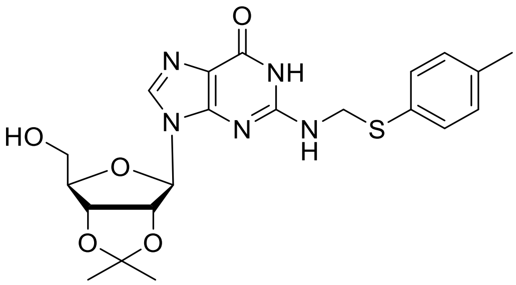

The reaction was carried out slightly modified to Bridson *et al.*.[10]

2',3'-O-isopropylideneguanosine (2.50 g, 7.73 mmol, 1 eq.) and p-thiocresole (1.25 g, 10.1 mmol, 1.3 eq.) were suspended in EtOH (60.0 mL). Acetic acid (1.9 mL) and formaldehyde (2.0 mL) were added to the suspension and the mixture was heated to 90°C. After 14 h the solvent was removed *in vacuo* and the crude product was washed with EtOH to afford the product as a white solid (2.75 g, 5.88 mmol, yield: 76%).

^{1}H NMR (400 MHz, DMSO-d_6) δ = 10.79 (s, 1H, $N_{Ar}H$), 7.95 (s, 1H, HC8), 7.41 – 7.31 (m, 2H, J = 8.0 Hz, HC_{Ar}), 7.17 (d, J = 8.0 Hz, 2H, HC_{Ar}), 6.01 (d, J = 2.6 Hz, 1H, HC1'), 5.36 (dd, J = 6.3, 2.7 Hz, 1H, HC2'), 5.02 (t, J = 5.5 Hz, 1H, OH), 4.95 (dd, J = 6.3, 3.0 Hz, 1H, HC3'), 4.84 (h, J = 6.7, 6.3 Hz, 2H, CH_2C_{Ar}), 4.13 (td, J = 5.5, 3.0 Hz, 1H, HC4'), 3.54 (hept, J = 5.7 Hz, 2H, HC5'), 2.28 (s, 3H, $C_{Ar}CH_3$), 1.54 (s, 3H, CH_3), 1.35 (s, 3H, CH_3). **^{13}C NMR** (101 MHz, DMSO-d_6) δ = 154.14 (C6), 151.76 (C2), 149.98 (C4), 137.49 (C8), 136.99 ($C_{Ar}CH_3$), 136.31

($C_{Ar}S$), 131.43 ($C_{Ar}S$), 130.24 (2C, C_{Ar}), 118.02 (C5), 113.49 ($C(CH_3)_2$), 89.17 (C1’), 87.10 (C4’), 83.75 (C2’), 81.74 (C3’), 61.99 (C5’), 46.71 (CH_2C_{Ar}), 27.41 (CH_3), 25.52(CH_3), 21.04 ($C_{Ar}CH_3$). **HRMS** (ESI+): calc. for $[C_{21}H_{26}N_5O_5S]^+$ 460.1649, found: 460.1650 $[M+H]^+$.

9-((3aR,4R,6R,6aR)-6-(hydroxymethyl)-2,2-dimethyltetrahydrofuro[3,4-d][1,3]dioxol-4-yl)-2-(methylamino)-1,9-dihydro-6H-purin-6-one (16)

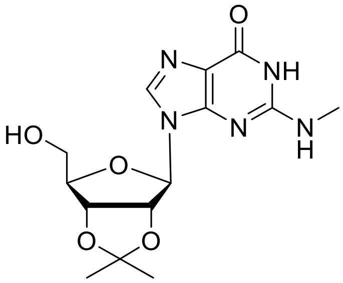

The reaction was carried out slightly modified to Bridson *et al.*.[10]

Compound **15** (0.65 g, 1.41 mmol, 1 eq.) was dissolved in DMSO (9.0 mL). $NaBH_4$ (0.11 g, 2.81 mmol, 2 eq.) was added and the mixture was heated to 100°C. After cooling to rt the mixture was purified by column chromatography (DCM : MeOH, 9:1) to afford the product as a white solid (0.41 g, 1.22 mmol, yield: 86%).

^{1}H NMR (400 MHz, DMSO-d_6) δ = 10.80 (s, 1H, $N_{Ar}H$), 7.90 (s, 1H, HC8), 6.46 (m, 1H, $NHCH_3$), 6.00 (d, *J* = 2.6 Hz, 1H, HC1’), 5.31 (dd, *J* = 6.2, 2.6 Hz, 1H, HC2’), 5.00 (t, *J* = 5.5 Hz, 1H, OH), 4.95 (dd, *J* = 6.2, 3.0 Hz, 1H, HC3’), 4.12 (td, *J* = 5.5, 3.0 Hz, 1H, HC4’), 3.53 (m, 2H, HC5’), 2.82 (d, *J* = 4.6 Hz, 3H, NCH_3), 1.53 (s, 3H, CH_3), 1.32 (s, 3H, CH_3). **^{13}C NMR** (101 MHz, DMF-d_7) δ = 157.49 (C2), 154.08 (C6), 150.98 (C4), 136.86 (C8), 117.84 (C5), 113.59 ($C(CH_3)_2$), 89.79 (C1’), 87.52 (C4’), 84.22 (C2’), 82.17 (C3’), 62.50 (C5’), 27.88 (NCH_3), 27.03 (CH_3), 25.06 (CH_3). **HRMS** (ESI+): calc. for $[C_{14}H_{20}N_5O_5]^+$ 338.1457, found: 338.1459 $[M+H]^+$.

9-((2R,3R,4S,5R)-3,4-dihydroxy-5-(hydroxymethyl)tetrahydrofuran-2-yl)-2-(methyl-amino)-1,9-dihydro-6H-purin-6-one (m^{2}G, 17)

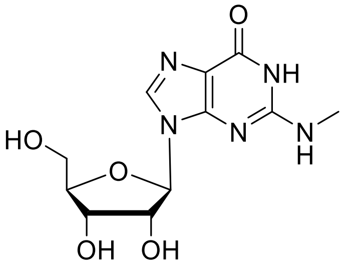

The reaction was carried out slightly modified to Ubiali *et al.*.[11]

The protected nucleoside **16** (0.28 g, 0.83 mmol, 1 eq.) was dissolved in H_2O (25.0 mL) and TFA (25.0 mL). After stirring for 1.5 h the solvent was removed in vacuo. The residue was suspended in Aceton and H_2O. The solvent was removed in vacuo and the raw product was recrystalized in EtOH/ H_2O to yield the product as a white solid (0.16 mg, 0.54 mmol, yield: 65%).

^{1}H-NMR (400 MHz, CD_3OD) δ = 10.77 (s, 1H, $N_{Ar}H$), 7.95 (s, 1H, HC8), 6.28 (d, ^3J = 5.0 Hz, 1H, NH), 5.73 (d, ^3J = 6.0 Hz, 1H, HC1’), 4.52 (t, J = 5.5 Hz, 1H, HC2’), 4.12 (dd, J = 5.1, 3.4 Hz, 1H, HC3’), 3.88 (d, J = 3.9 Hz, 1H, HC4’), 3.75 – 3.45 (m, 2H, HC5’), 2.81 (d, ^3J = 4.7 Hz, 3H, NCH_3). **^{13}C-NMR** (101 MHz, CD_3OD) δ = 156.77 (C6), 153.29 (C2), 150.97 (C4), 136.15 (C8), 116.65 (C5), 86.79 (C1’), 85.31 (C4’), 73.39 (C2’), 70.55 (C3’), 61.60 (C5’), 27.74 (NCH_3). **HRMS** (ESI+): calc. for $[C_{11}H_{16}N_5O_4]^+$ 298.1146, found: 298.1142 $[M+H]^+$.

(2R,3R,4R,5R)-2-(acetoxymethyl)-5-(6-chloro-2-(methylthio)-9H-purin-9-yl)tetrahydro-furan-3,4-diyl diacetate (18)

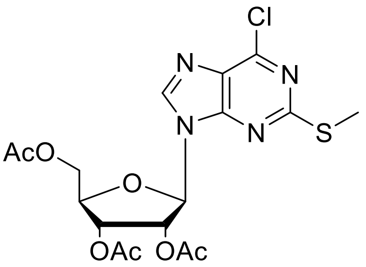

The reaction was carried out slightly modified to Kierzek *et al.*.[12]

Nucleoside **8** (2.00 g, 4.68 mmol, 1 eq.) was dissolved in ACN (3.5 mL). Dimethylsulfide (4.40 g, 46.8 mmol, 10 eq.) and isopentenylnitrite (0.95 g, 9.35 mmol, 2 eq.) were added to the solution and the mixture was heated to 65°C. After 1 h, 2 more equivlents of isopentenylnitrite (0.95 g, 9.35 mmol, 2 eq.) were added. After 30 min the solvent was removed *in vacuo* and the crude product was purified by column chromatography (DCM : MeOH, 100:1) to afford the product as a yellow foam (1.59 g, 3.47 mmol, yield: 74%).

^{1}H-NMR (400 MHz, DMSO-d_6) δ = 8.70 (s, 1H, HC8), 6.30 (d, J = 4.1 Hz, 1H, HC1‘), 6.04 (dd, J = 6.0, 4.2 Hz, 1H), 5.80 – 5.62 (m, 1H, HC2‘), 4.48 – 4.17 (m, 4H, HC3‘, HC4‘, HC5‘), 2.63 (s, 3H, SCH_3), 2.11 (s, 3H, $COCH_3$), 2.08 (s, 3H, $COCH_3$), 1.96 (s, 3H, $COCH_3$). **^{13}C-NMR** (151 MHz, $CDCl_3$) δ = 170.2 (CH_3CO), 169.4 (CH3CO), 169.2 (CH3CO), 167.4 (C2), 151.9 (C6), 151.3 (C4), 142.1 (C8), 129.1 (C5), 87.0 (C1´), 80.0 (C4´), 72.9 (C2´), 70.1 (C3´), 62.7 (C5´), 20.7 (CH_3CO), 20.5 (CH_3CO), 20.4 (CH_3CO), 14.8 (SCH_3). **HRMS** (ESI^+): calc. for $[C_{17}H_{20}ClN_4O_7S]^+$ 459.0738, found: 459.0736 $[M+H]^+$.

(2R,3R,4S,5R)-2-(6-amino-2-(methylthio)-9H-purin-9-yl)-5-(hydroxymethyl)tetra-hydrofuran-3,4-diol (ms²A, 19)

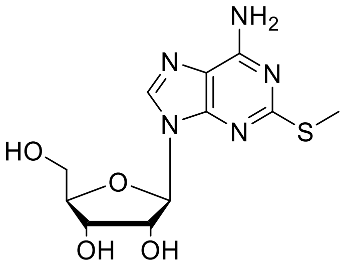

The reaction was carried out slightly modified to Yamazaki *et al.*.[7,8]

Nucleoside **18** (50.0 mg, 110 µmol, 1 eq.) was condensed into a pressure tube with ammonia and stirrerd for 24 h at rt. The solvent was removed and the crude product was recrystallized in EtOH to afford the product as a white solid (26.0 mg, 79.4 µmol, yield: 73%).

^{1}H-NMR (400 MHz, DMSO-d_6) δ = 8.23 (s, 1H, HC8), 7.38 (s, 2H, NH_2), 5.83 (d, J = 6.0 Hz, 1H, HC1‘), 5.45 (d, J = 6.2 Hz, 1H, OH), 5.21 (d, J = 4.8 Hz, 1H, OH), 5.03 (t, J = 5.6 Hz, 1H, OH), 4.62 (q, J = 5.8 Hz, 1H, HC2‘), 4.15 (td, J = 4.9, 3.3 Hz, 1H, HC3‘), 3.91 (q, J = 4.1 Hz, 1H, HC4‘), 3.70 – 3.49 (m, 2H, HC5‘), 2.47 (s, 3H, SCH_3). **^{13}C-NMR** (101 MHz, DMSO) δ = 164.62 (C2), 155.90 (C6), 150.61 (C4), 139.24 (C8), 117.32 (C5´), 87.68 (C1´), 85.89 (C4´), 73.67 (C2´), 70.96 (C3´), 62.04 (C5´), 14.13 (SCH_3). **HRMS** (ESI$^+$): calc. for $[C_{11}H_{16}N_5O_4S]^+$ 314.0915, found: 314.0918 $[M+H]^+$.

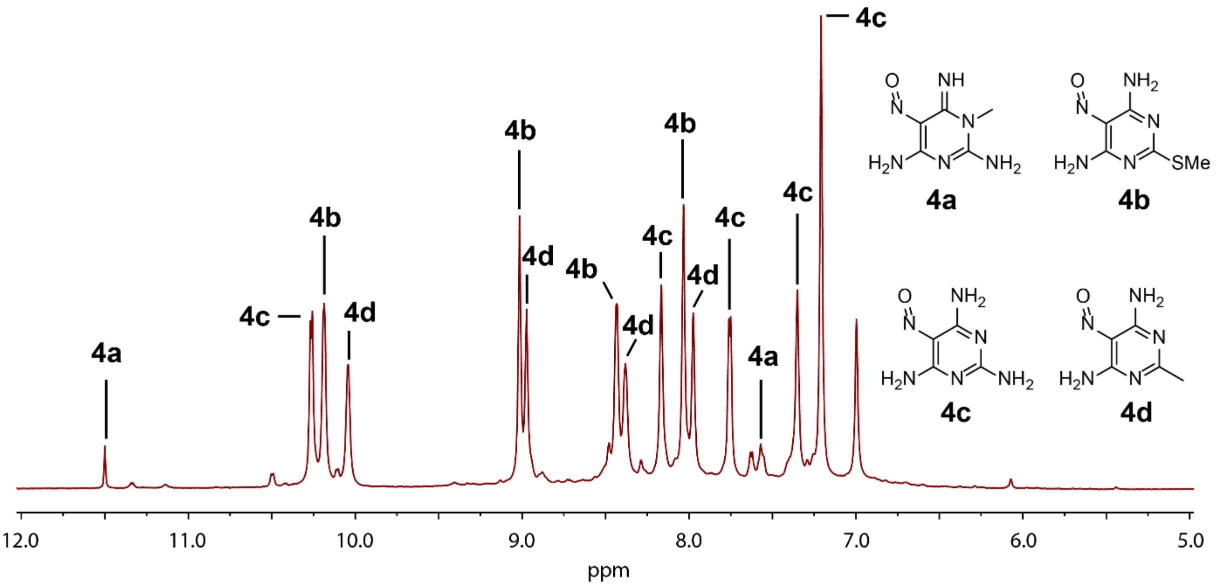

Supplementary Figure 1: Simultaneous formation of nitroso-pyrimidines **4a-d**. ^{1}H-NMR after salts containing **2a-d** and **3** were mixed and heated with a temperature gradient (1 °C/5 min, from 100-160 °C).

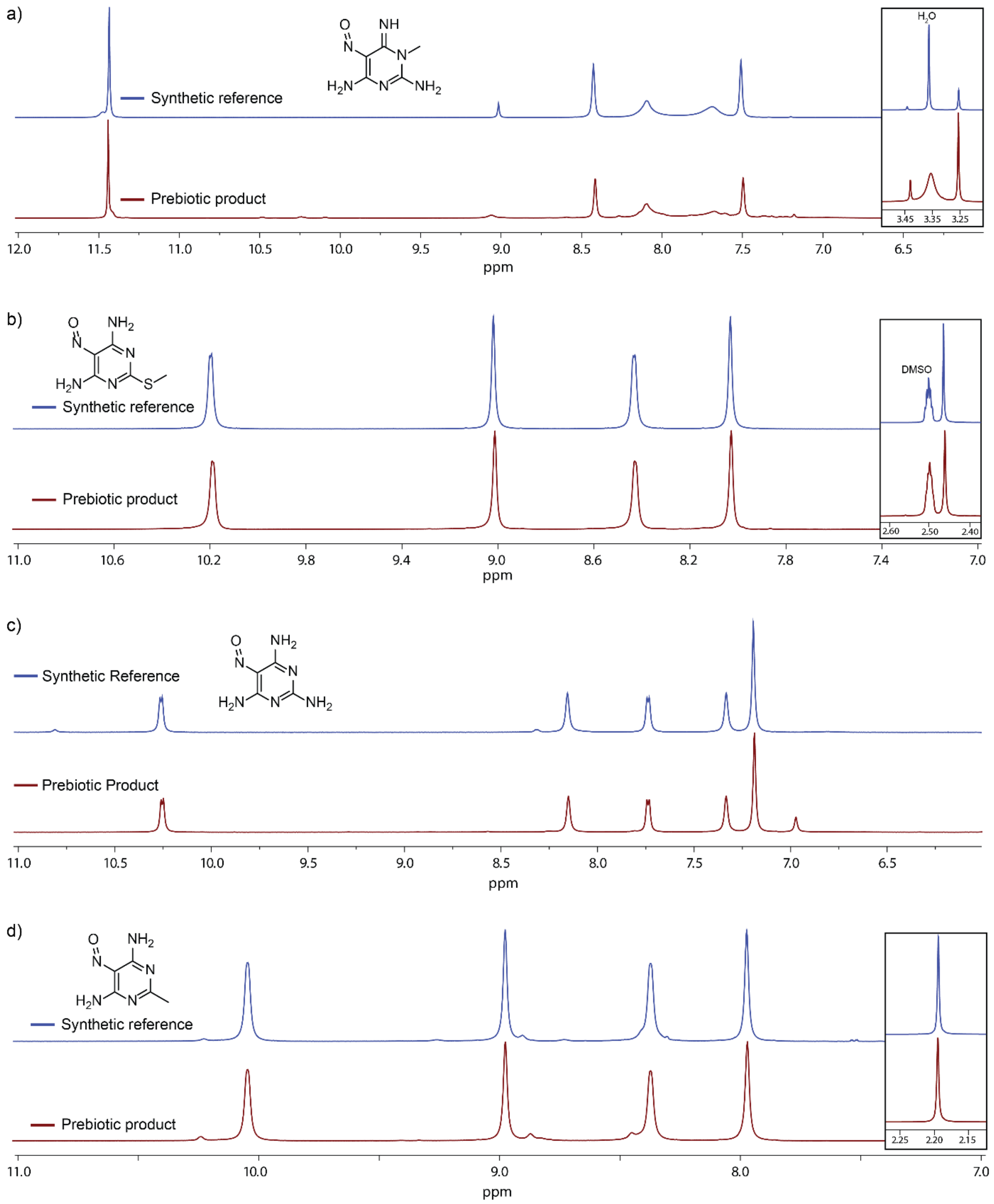

Supplementary Figure 2: ^{1}H-NMR comparism of prebiotic and synthetic product **4a-d**. The spectroscopic data for the prebiotic formation of nitroso-pyrimidines **4a-d** after physical enrichment shows very clean products, when compared with synthetic standards.

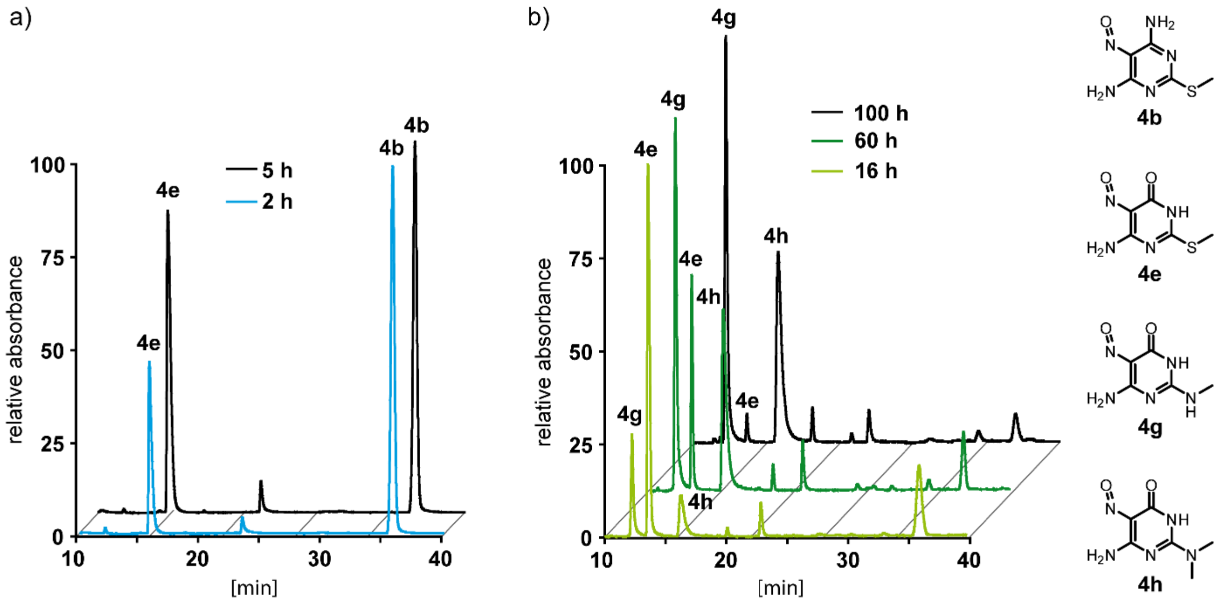

Supplementary Figure 3: One-pot conversion of **4b** to **4g** and **4h** by pH change. The figure shows the UV-chromatogram (at 325 nm) from LC/MS measurements of the hydrolysis of **4b** to **4e** and subsequent aminolysis to **4g** and **4h**. a) hydrolysis of **4b** to **4e** after 2 h and 5 h in 3 M HCl at room temperature containing methylamine hydrochloride (300 mM) and dimethylamine hydrochloride (100 mM). b) the pH of the reaction mixture was carefully adjusted after 5 h with Na_2CO_3 to pH ~9-10. Under basic conditions **4b** mainly precipitates whereas **4e** stays in solution. The reaction mixture is shown after 16 h, 60 h, and 100 h. **4e** is almost completely converted due to nucleophilic substitution into **4g** and **4h** after ~4 days.

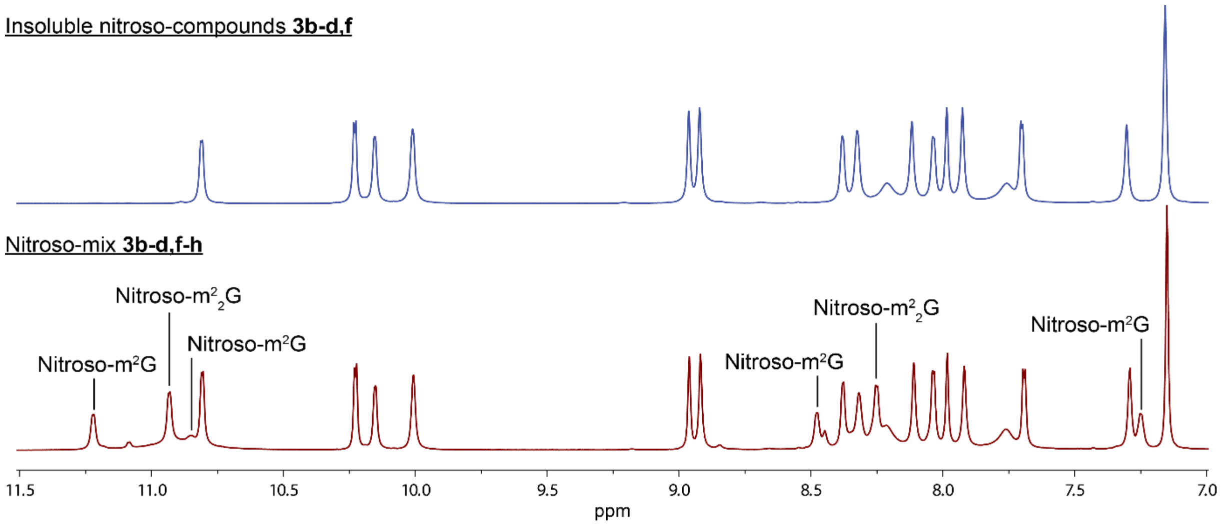

Supplementary Figure 4: Separation of nitroso-pyrimidines from a mixture (**4b-d,f-h**). Even though **4i** is soluble under basic conditions, this compound was excluded due to it being insufficiently soluble for NMR measurements. Procedure: a mixture containing 12 mg each of **4b-d,f-h** was treated with 15% NH_4OH (4 ml). The insoluble compounds were filtered off and dried. NMR of the insoluble compounds shows that compound **4g** (Nitroso-m^{2}G) and **4h** (Nitroso-m$^2_2$G) are absent from the mixture.

Supplementary Table 1: crystal data of 1-methylguanidine (**2a**) salt of (hydroxyimino)-malononitrile (**3**)

CCDC number	1574226
net formula	$C_5H_8N_6O$
M_r/g mol^{-1}	168.17
crystal size/mm	0.100 × 0.030 × 0.030
T/K	100.(2)
radiation	MoKα
diffractometer	'Bruker D8 Venture TXS'
crystal system	orthorhombic
space group	'P n a 21'
a/Å	16.4092(14)
b/Å	13.0181(10)
c/Å	3.7470(3)
α/°	90
β/°	90
γ/°	90
V/Å^3	800.42(11)
Z	4
calc. density/g cm^{-3}	1.396
μ/mm^{-1}	0.106
absorption correction	Multi-Scan
transmission factor range	0.8400–0.9705
refls. measured	6118
R_{int}	0.0560
mean σ(I)/I	0.0514
θ range	3.367–26.345
observed refls.	1447
x, y (weighting scheme)	0.0441, 0.3047
hydrogen refinement	H(C) constr, H(N) refall
Flack parameter	0.5
refls in refinement	1597
parameters	130
restraints	1
$R(F_{obs})$	0.0417
$R_w(F^2)$	0.1057
S	1.083
shift/error$_{max}$	0.001
max electron density/e Å^{-3}	0.187
min electron density/e Å^{-3}	−0.240

Supplementary Table 2: crystal data of methylthioamidine (**2b**) salt of (hydroxyimino)-malononitrile (**3**)

CCDC number	1574223
net formula	$C_5H_7N_5OS$
M_r/g mol^{-1}	185.22
crystal size/mm	0.100 × 0.090 × 0.050
T/K	100.(2)
radiation	MoKα
diffractometer	'Bruker D8 Venture TXS'
crystal system	triclinic
space group	'P -1'
a/Å	7.5580(4)
b/Å	9.9840(5)
c/Å	12.0318(7)
α/°	73.888(2)
β/°	86.151(2)
γ/°	80.423(2)
V/Å^3	859.88(8)
Z	4
calc. density/g cm^{-3}	1.431
μ/mm^{-1}	0.337
absorption correction	Multi-Scan
transmission factor range	0.8536–0.9705
refls. measured	8943
R_{int}	0.0247
mean σ(*I*)/*I*	0.0344
θ range	3.207–27.485
observed refls.	3268
x, y (weighting scheme)	0.0269, 0.3770
hydrogen refinement	H(C) constr, H(N) refall
refls in refinement	3886
parameters	251
restraints	1
$R(F_{obs})$	0.0311
$R_w(F^2)$	0.0787
S	1.070
shift/error$_{max}$	0.001
max electron density/e Å^{-3}	0.265
min electron density/e Å^{-3}	−0.255

Supplementary Table 3: crystal data of guanidine (**2c**) salt of (hydroxyimino)malononitrile (**3**)

CCDC number	1574225
net formula	$C_4H_6N_6O$
M_r/g mol^{-1}	154.15
crystal size/mm	0.100 × 0.060 × 0.040
T/K	100.(2)
radiation	MoKα
diffractometer	'Bruker D8 Venture TXS'
crystal system	orthorhombic
space group	'F d d 2'
a/Å	21.2099(12)
b/Å	33.2970(18)
c/Å	3.8794(2)
α/°	90
β/°	90
γ/°	90
V/Å^3	2739.7(3)
Z	16
calc. density/g cm^{-3}	1.495
μ/mm^{-1}	0.117
absorption correction	Multi-Scan
transmission factor range	0.9133–0.9705
refls. measured	10489
R_{int}	0.0381
mean σ(I)/I	0.0222
θ range	3.843–26.355
observed refls.	1322
x, y (weighting scheme)	0.0310, 7.2954
hydrogen refinement	refall
Flack parameter	0.5
refls in refinement	1384
parameters	124
restraints	2
$R(F_{obs})$	0.0360
$R_w(F^2)$	0.0926
S	1.158
shift/error$_{max}$	0.001
max electron density/e Å^{-3}	0.324
min electron density/e Å^{-3}	−0.189

Supplementary Table 4: crystal data of acetamidine (**2d**) salt of (hydroxyimino)-malononitrile (**3**)

CCDC number	1574224
net formula	$C_5H_7N_5O$
M_r/g mol^{-1}	153.16
crystal size/mm	0.080 × 0.050 × 0.040
T/K	100.(2)
radiation	MoKα
diffractometer	'Bruker D8 Venture TXS'
crystal system	triclinic
space group	'P -1'
a/Å	6.4976(3)
b/Å	6.9460(4)
c/Å	9.2031(5)
α/°	100.345(2)
β/°	94.349(2)
γ/°	113.040(2)
V/Å^3	371.08(3)
Z	2
calc. density/g cm^{-3}	1.371
μ/mm^{-1}	0.104
absorption correction	Multi-Scan
transmission factor range	0.6396–0.9705
refls. measured	3842
R_{int}	0.0569
mean σ(*I*)/*I*	0.0765
θ range	3.273–27.481
observed refls.	1389
x, y (weighting scheme)	0.0499, 0.1265
hydrogen refinement	H(C) constr, H(N) refall
refls in refinement	1673
parameters	117
restraints	0
$R(F_{obs})$	0.0520
$R_w(F^2)$	0.1361
S	1.079
shift/error$_{max}$	0.001
max electron density/e Å^{-3}	0.373
min electron density/e Å^{-3}	−0.289

Supplementary References

1 Brown, D. J. & Jacobsen, N. W. 612. Pyrimidine reactions. Part IV. The methylation of 2,4- and 4,5-diaminopyrimidine and related compounds. *J. Chem. Soc.*, 3172-3179 (1962).

2 Chang, L. *et al.* Imidazopyridine- and Purine-Thioacetamide Derivatives: Potent Inhibitors of Nucleotide Pyrophosphatase/Phosphodiesterase 1 (NPP1). *J. Med. Chem.* **57**, 10080-10100 (2014).

3 Robins, M. J. & Uznański, B. Nucleic acid related compounds. 33. Conversions of adenosine and guanosine to 2, 6-dichloro, 2-amino-6-chloro, and derived purine nucleosides. *Can. J. Chem.* **59**, 2601-2607 (1981).

4 Reiter, V. *Synthese von eukaryotischen RNA-Modifikationenund Quantifizierung nicht kanonischer Nukleoside sowie Untersuchungen zu deren Biosynthese*, Thesis, LMU München, (2013).

5 Matsuda, A. *et al.* Nucleosides and nucleotides. 103. 2-Alkynyladenosines: a novel class of selective adenosine A2 receptor agonists with potent antihypertensive effects. *J. Med. Chem.* **35**, 241-252 (1992).

6 Yamazaki, A. *et al.* Synthesis of Thioinosine and Thio-AICA-riboside Analogs. *Chem. Pharm. Bull.* **21**, 692-696 (1973).

7 Davoll, J. & Lowy, B. A. Some Synthetic Analogs of the Natural Purine Nucleosides1. *J. Am. Chem. Soc.* **74**, 1563-1566 (1952).

8 Yamazaki, A., Kumashiro, I. & Takenishi, T. Synthesis of 2-methyladenosine and its 5'-phosphate. *J. Org. Chem.* **33**, 2583-2586 (1968).

9 Vorbrüggen, H., Krolikiewicz, K. & Bennua, B. Nucleoside syntheses, XXII1) Nucleoside synthesis with trimethylsilyl triflate and perchlorate as catalysts. *Chem. Ber.* **114**, 1234-1255 (1981).

10 Bridson, P. K. & Reese, C. B. A novel method for the methylation of heterocyclic amino groups. Conversion of guanosine into its 2-N-methyl-and 2-N,2-N-dimethyl derivatives. *Bioorg. Chem.* **8**, 339-349 (1979).

11 Ubiali, D. *et al.* Production, characterization and synthetic application of a purine nucleoside phosphorylase from Aeromonas hydrophila. *Adv. Synth. Catal.* **354**, 96-104 (2012).

12 Kierzek, E. & Kierzek, R. The thermodynamic stability of RNA duplexes and hairpins containing N6-alkyladenosines and 2-methylthio-N6-alkyladenosines. *Nucleic Acids Res.* **31**, 4472-4480 (2003).
